# Identifying adverse reactions following COVID-19 vaccination in Korea using data from active surveillance: a text mining approach

**DOI:** 10.4178/epih.e2025034

**Published:** 2025-06-30

**Authors:** Hye Ah Lee, Bomi Park, Chung Ho Kim, Yeonjae Kim, Hyunjin Park, Seunghee Jun, Hyelim Lee, Seunghyun Lewis Kwon, Yesul Heo, Hyungmin Lee, Hyesook Park, COVID-19 Vaccine Safety Research Committee

**Affiliations:** 1 Clinical Trial Center, Ewha Womans University Mokdong Hospital, Seoul, Korea; 2 COVID-1 9 Vaccine Safety Research Center, Seoul, Korea; 3 Department of Preventive Medicine, Chung-Ang University College of Medicine, Seoul, Korea; 4 Department of Preventive Medicine, Ewha Womans University College of Medicine, Seoul, Korea; 5 Graduate Program in System Health Science and Engineering, Ewha Womans University, Seoul, Korea; 6 Division of Immunization Services, Korea Disease Control and Prevention Agency, Cheongju, Korea; 7 KDI School of Public Policy and Management, Sejong, Korea; 8 Division of Immunization Policy, Korea Disease Control and Prevention Agency, Cheongju, Korea

**Keywords:** Adverse reactions, Data mining, Pharmacovigilance, Safety, Vaccination

## Abstract

**OBJECTIVES:**

Unstructured text data collected through vaccine safety surveillance systems can identify previously unreported adverse reactions and provide critical information to enhance these systems. This study explored adverse reactions using text data collected through an active surveillance system following coronavirus disease 2019 (COVID-19) vaccination.

**METHODS:**

We performed text mining on 2,608 and 2,054 records from 2 survey seasons (2023-2024 and 2024-2025), in which participants reported health conditions experienced within 7 days of vaccination using free-text responses. Frequency analysis was conducted to identify key terms, followed by subgroup analyses by sex, age, and concomitant influenza vaccination. In addition, semantic network analysis was used to examine terms reported together.

**RESULTS:**

The analysis identified several common (≥1%) adverse events, such as respiratory symptoms, sleep disturbances, lumbago, and indigestion, which had not been frequently noted in prior literature. Moreover, less frequent (≥0.1 to <1.0%) adverse reactions affecting the eyes, ears, and oral cavity were also detected. These adverse reactions did not differ significantly in frequency based on the presence or absence of simultaneous influenza vaccination. Co-occurrence analysis and estimation of correlation coefficients further revealed associations between frequently co-reported symptoms.

**CONCLUSIONS:**

This study utilized text mining to uncover previously unrecognized adverse reactions associated with COVID-19 vaccination, thereby broadening our understanding of the vaccine’s safety profile. The insights obtained may inform future investigations into vaccine-related adverse reactions and improve the processing of text data in surveillance systems.

## GRAPHICAL ABSTRACT

[Fig f4-epih-47-e2025034]

## Key Message

• This study used text mining of self-reported symptoms following COVID-19 vaccination to identify previously unrecognized adverse reactions.

• Our findings enhance the understanding of vaccine side effects and provide valuable insights for future research and surveillance system improvement.

## INTRODUCTION

To address the unprecedented coronavirus disease 2019 (COVID-19) pandemic and mitigate its public health impact, the development, approval, and distribution of safe and effective vaccines were rapidly accelerated [1]. Consequently, many countries have established and implemented monitoring systems to ensure COVID-19 vaccine safety [1-3]. In Korea, both a web-based passive surveillance system and a text message-based active surveillance system have been deployed for vaccine safety monitoring [4,5]. Unlike systems relying solely on electronic health records, active surveillance proactively collects self-reported symptoms or newly developed health conditions from vaccinated individuals during a predefined period, thereby enabling near real-time safety monitoring [3]. These systems have made substantial contributions to identifying unexpected or undesirable adverse reactions, providing a basis for anticipating adverse events related to vaccines for future COVID-19 variants or other viral infections.

Since its emergence, severe acute respiratory syndrome coronavirus-2 (SARS-CoV-2) has undergone continuous and significant evolution, necessitating ongoing vaccination with updated COVID-19 vaccines as a key public health strategy [6]. The Korea Disease Control and Prevention Agency (KDCA) releases annual vaccination plans to prevent the spread of COVID-19. Beginning with the 2023-2024 season, this plan has recommended concurrent administration of COVID-19 and seasonal influenza vaccines for individuals aged 65 years and older [7-9]. Numerous surveillance studies have evaluated vaccine safety and contributed to reducing public hesitancy [10-12]. Monitoring through spontaneous reporting systems can rapidly identify emergent safety signals and novel adverse reactions of concern [1]. In Korea, as the COVID-19 vaccine program was integrated with the influenza vaccination campaign, an active, text message-based surveillance system was introduced. This system encouraged participants to report any adverse reactions experienced—beyond those typically captured within the first 7 days after vaccination—via text message. By enhancing the questionnaire, this approach broadened the scope of adverse event investigation [13]. Text-based data thus offer valuable insights into adverse reactions that may not have been previously reported. Text mining methods can extract hidden knowledge by identifying patterns in large volumes of unstructured text [14]. While several studies have assessed adverse reactions to the coadministration of COVID-19 and influenza vaccines [15,16], ongoing vigilance in monitoring adverse reactions in the general population remains vital for comprehensive safety assessment.

Analyses of social media data, such as those from Twitter, related to COVID-19, have often focused on tracking the evolution of emotions and sentiments throughout the pandemic [17-19], as well as on the analysis of topics and information sharing [20-22]. In the context of COVID-19 vaccination, text analysis has examined reasons for vaccine hesitancy [23], and sentiment analysis has been conducted regarding the vaccine itself [24]. Methods such as sentiment analysis and topic modeling have been used to investigate public perceptions. However, few studies have applied text analysis specifically to identify potential adverse reactions.

Therefore, in this study, we conducted a text mining analysis to identify potential adverse reactions following COVID-19 vaccination using records collected across 2 seasons (2023-2024 and 2024-2025). Because vaccine types varied between seasons, we analyzed each season separately and further examined whether adverse reaction reporting differed according to concomitant influenza vaccination.

## MATERIALS AND METHODS

### Data source

This study used anonymized data collected by the KDCA. The KDCA conducted active surveillance through the Immunization Registry Information System (IRIS) following administration of the COVID-19 XBB.1.5 vaccine during the 2023-2024 season and the COVID-19 JN.1 vaccine during the 2024-2025 season [25]. Further details on the surveillance system are described elsewhere [4,5]. The 2023-2024 vaccination campaign, which began on October 19, 2023, initially targeted individuals aged 65 years and older, immunocompromised individuals aged 12 to 64, and residents and workers in vulnerable facilities such as nursing hospitals and nursing homes. The campaign was subsequently expanded to include all individuals aged 12 to 64 beginning November 1 [7]. The 2024-2025 vaccination campaign, which commenced on October 11, 2024, focused on individuals aged 65 years and older, immunocompromised patients, hospitalized individuals, and residents and workers at vulnerable facilities [8]. A text-message (short message service, SMS)-based surveillance survey was administered to vaccine recipients who consented to participate via smartphone, monitoring adverse reactions and health status for 7 days post-vaccination. Surveillance was planned to collect data from 10,000 respondents per vaccine manufacturer [12].

Collected data included sex, age, vaccination date, vaccine manufacturer, and information regarding concurrent influenza vaccination. Post-vaccination outcome data comprised any health issues, fever, adverse reactions, interference with daily activities, and healthcare facility visits. Questionnaires on adverse reactions were divided into categories for local and systemic reactions, with responses captured through closed-ended questions. Respondents could report multiple adverse reactions. Additionally, an open-ended question allowed participants to describe any other local or systemic health issues experienced after vaccination in free-text form. For this study, we analyzed text data obtained from the open-ended question. Newly identified adverse reactions were defined as symptoms not included in official national reports [26] or in the predefined local and systemic reactions listed in the questionnaire.

The first survey period (2023-2024 season) included data on adverse reactions in individuals who received the COVID-19 vaccine (10,099 Pfizer-BioNTech and 10,083 Moderna) between October 19, 2023, and November 6, 2023, and who responded to text messages at least once within 7 days post-vaccination. The second survey period (2024-2025 season) included data from individuals vaccinated between October 11, 2024, and November 30, 2024 (10,002 Pfizer-BioNTech, 10,003 Moderna, and 2,906 Novavax). Thus, for text mining analysis, the analytical dataset consisted of 2,608 records (i.e., total submissions, including multiple entries from the same individual) from 1,864 individuals in the first period and 2,054 records from 1,515 individuals in the second period. Data on concurrent COVID-19 and seasonal influenza vaccinations were also obtained from the IRIS. Variables including sex, age, vaccine manufacturer, and concurrent influenza vaccination were considered in the analysis.

### Data preprocessing for text mining

Text preprocessing is essential in text mining to remove noise and extract meaningful information [27]. The specific procedures used in this study are described in the Supplementary Material 1.

### Data analysis

#### Descriptive analysis

A basic descriptive analysis was conducted on 1,864 (survey period 1) and 1,515 (survey period 2) COVID-19 vaccine recipients. Categorical variables are presented as frequencies and percentages, while continuous variables are reported as means with standard deviations and medians with interquartile ranges. We also evaluated the impact of concurrent administration of the seasonal influenza vaccine on basic characteristics using the chi-square test, t-test, and Mann-Whitney U test.

#### Word frequencies

The frequency of adverse reaction-related words was categorized as very common (≥10%), common (≥1 to <10%), or uncommon (≥0.1 to <1.0%) [28]. In addition, the daily reporting percentage for adverse reactions was calculated. Additional details are provided in the Supplementary Material 1.

#### Semantic network

To uncover patterns in the co-occurrence of adverse reactions, we conducted both co-occurrence and phi-coefficient analyses, visualizing the results to facilitate interpretation. In network analysis, linguistic units are represented as nodes, while relationships observed in actual language use are depicted as links. Keywords with high centrality are considered core terms and can be characterized by degree centrality, betweenness centrality, or closeness centrality. Degree centrality reflects how directly a keyword is connected to other keywords. Betweenness centrality indicates the extent to which a node functions as an intermediary between other nodes. Closeness centrality is based on the distance between nodes and illustrates how close a node is to others. These centrality metrics can be quantified [29]. To examine the usage context of specific words, network analysis included terms such as “injection.”

The co-occurrence network visualized relationships using lines, with the thickness of each line representing the frequency of paired term appearances, regardless of order. The network visualized relationships among keywords reported more than ten times, with node size corresponding to degree centrality.

The phi-coefficient quantifies the degree to which pairs of terms co-occur relative to their individual frequencies and is equivalent to the correlation coefficient for binary variables [30]. For the correlation matrix, we visualized only term pairs with a correlation >0.1, with line thickness representing the phi-correlation coefficient. The Infomap algorithm was applied to group nodes, which were assigned the same color to denote group membership.

All analyses were performed using R version 4.3.1 (R Foundation for Statistical Computing, Vienna, Austria), with R packages including “KoNLP,” “tidyr,” “tidytext,” and “ggraph.”

### Ethics statement

All participants provided informed consent to be included in the surveillance database. The study protocol was reviewed and approved by the Institutional Review Board of Ewha Womans University (No. ewha-202401-0011-01).

## RESULTS

Among vaccinated individuals, 1,864 (9.2%) and 1,515 (6.6%) responded during the first and second survey periods, respectively, and were included in the analysis. In the first survey period, 65.7% of respondents were male, with a mean age of 68.11 years, and each participant reported adverse reactions an average of 1.4 times. Additionally, 38.2% (n=712) of respondents received the seasonal influenza vaccine concurrently. In the second survey period, 63.0% of the 1,515 respondents were male, with a mean age of 70.98 years. Each individual reported an average of approximately 1.4 adverse reactions, and 81.2% (n=1,230) received the seasonal influenza vaccine at the same time. Compared to those who received only the COVID-19 vaccine, individuals who received both COVID-19 and influenza vaccines concurrently had a higher proportion of persons over 65 years of age, and most received the Moderna vaccine (Table 1).

During the 2023-2024 season, a total of 1,864 individuals reported 2,608 adverse reactions via text messages following vaccination, while in the 2024-2025 season, 1,515 individuals reported a total of 2,054 adverse reactions. Adverse reactions were most frequently reported the day after vaccination, with reporting decreasing over time. There were no significant differences in the percentage of adverse reactions reported within the first 7 days after vaccination between groups stratified by concurrent influenza vaccination status (Supplementary Material 2).

Table 2 displays the terms reported by more than 1% of individuals who reported adverse reactions through text messages. The most frequently reported term was “body aches” (21.9%), with other cold-related symptoms also frequently cited. Sleep-related symptoms, such as “drowsiness” (2.5%) and “sleep disturbance” (1.7%), were also observed. Additionally, terms such as “chest tightness” (1.4%), “lumbago” (1.4%), “shortness of breath” (1.4%), “indigestion” (1.3%), “heart palpitations” (1.3%), and “loss of appetite” (1.1%) were noted. In the second survey period, “body aches” (18.5%) remained the most frequently reported term, while “lumbago” (1.5%), “drowsiness” (1.4%), and “indigestion” (1.3%) were also commonly reported.

Although the frequencies were classified as uncommon (<1.0%), eye-related adverse reactions such as “vision abnormality” (0.8%), “eye pain” (0.4%), and “dry eyes” (<0.1%) were documented. Ear-related reactions, including “tinnitus” (0.4%), “earache” (0.3%), and “ear fullness” (0.2%), were also reported. From the fifth day after vaccination, 4 individuals reported “stomatitis” (0.2%) as an adverse reaction. Other oral cavity-related adverse reactions included “gum pain” (0.2%), “dry mouth” (0.2%), “gum swelling” (0.1%), and “tooth pain” (0.1%). Additional reported symptoms included “hoarseness” (0.7%), “blushing” (0.6%), “increased blood pressure (BP)” (0.4%), and “brain fog” (0.4%). “Memory impairments” were reported by 3 individuals (0.2%), all of whom had received only the COVID-19 vaccine. In the second survey period, terms related to the eyes (“vision abnormality,” “eye fatigue,” “eyelid,” “blepharospasm”) and ears (“tinnitus,” “earache,” “ear fullness”) were again reported. Other reactions included “hoarseness” (0.9%), “blushing” (0.5%), “increased BP” (0.7%), and “brain fog” (0.5%) (Supplementary Material 3).

**Figure 1.**
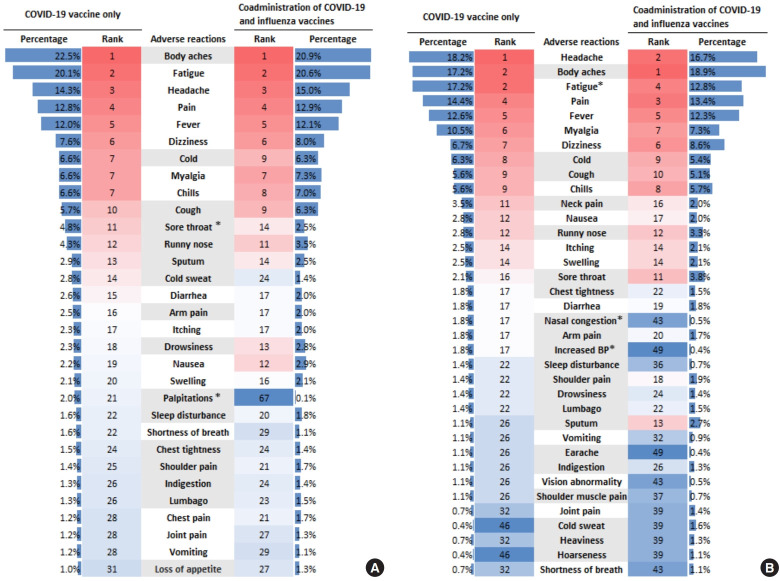
Differences in frequent terms for adverse reactions reported via text during the first 7 days following coronavirus disease 2019 (COVID-19) vaccination, according to with or without a concurrent influenza vaccine: (A) survey period 1 (October 19, 2023 to November 6, 2023) and (B) survey period 2 (October 11, 2024 to November 30, 2024). Gray background indicates newly identified adverse reactions via text analysis. *p<0.05.

Terms related to frequently reported adverse reactions showed differences in ranking between groups, but the reported percentages were generally similar. However, some terms differed between groups: “sore throat” and “palpitations” were reported than when both COVID-19 and influenza vaccines were given together. In the second survey period, the frequencies of “nasal congestion” and “increased BP” also differed (Figure 1). Regarding differences in reporting by sex, the terms “headache,” “cold sweat,” “shortness of breath,” “vomiting,” and “indigestion” were reported more often by females than males. In the second survey period, “neck pain,” “nausea,” and “joint pain” were more frequently reported by women, whereas “fatigue,” “drowsiness,” and “sleep disturbance” were more commonly reported by males (Supplementary Material 4). The frequency of reported “joint pain” varied across age groups and was higher in younger participants (Supplementary Material 5).

**Figure 2.**
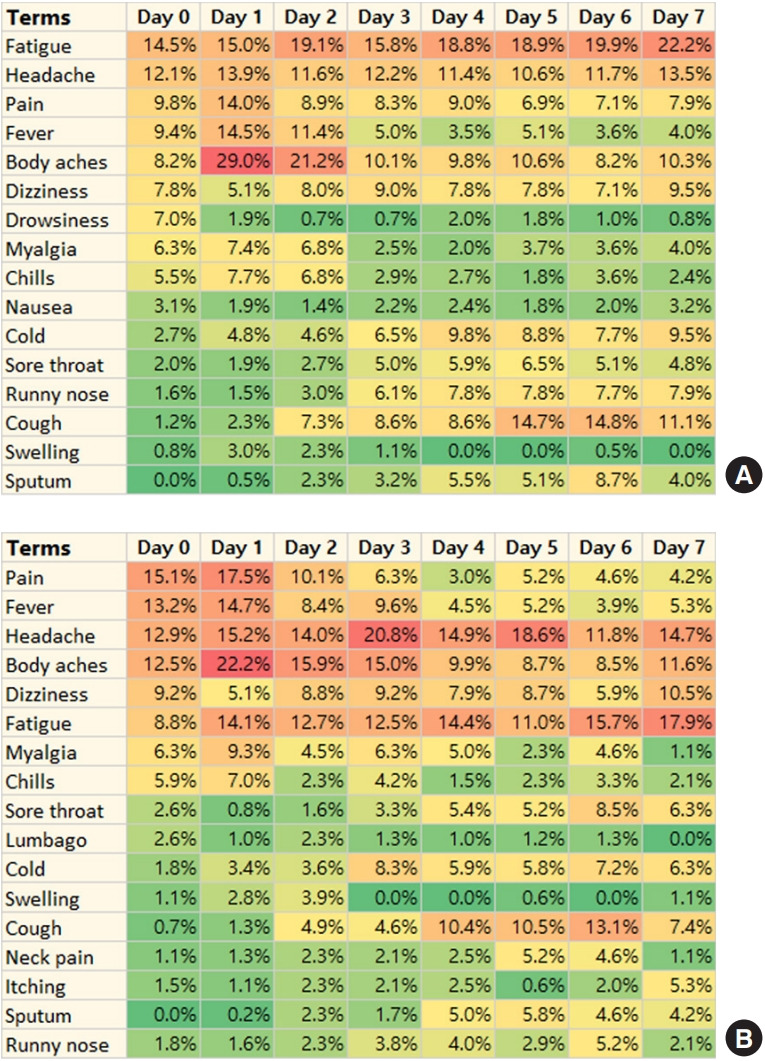
Percentages of daily reports of frequently reported terms during the first 7 days following coronavirus disease 2019 vaccination according to survey period: (A) survey period 1 (October 19, 2023 to November 6, 2023) and (B) survey period 2 (October 11, 2024 to November 30, 2024). The percentages were calculated using the frequency of reported terms, focusing on those who provided adverse reactions via text on each day.

The reporting percentages for specific, frequently reported terms either remained constant, decreased, or increased over the 7 days following vaccination. The term “body aches” was most frequently reported the day after vaccination and tended to decline thereafter. In contrast, terms such as “sputum,” “cough,” “runny nose,” “sore throat,” and “cold” tended to increase as time progressed post-vaccination. Similar trends were seen in the second survey period (Figure 2).

**Figure 3.**
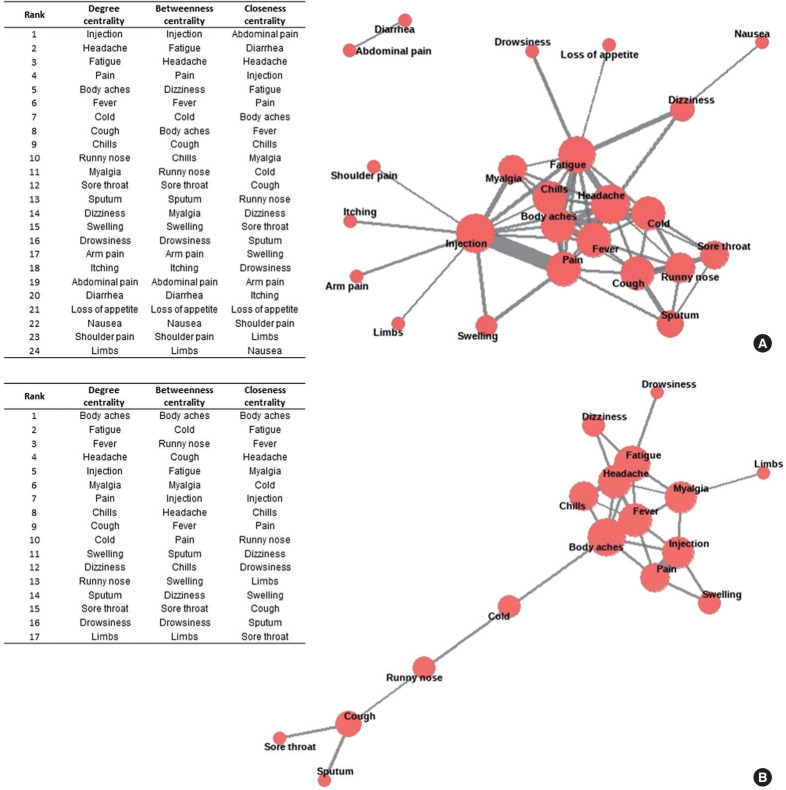
Co-occurrence network diagram for terms related to adverse reactions reported via text during the first 7 days following coronavirus disease 2019 vaccination according to survey period: (A) survey period 1 (October 19, 2023 to November 6, 2023) and (B) survey period 2 (October 11, 2024 to November 30, 2024). Node size indicates centrality and edge thickness indicates the frequency of term pairs. It is organized around key terms from pairs that have been reported more than 10 times.

Figure 3 presents the analysis of co-occurring terms. “injection” and “headache” were often reported alongside other terms. “Injection” was primarily reported together with “pain,” “myalgia,” and “swelling,” whereas “headache” was commonly co-reported with “fatigue,” “body aches,” and “dizziness.” This indicates that various systemic adverse reactions were frequently reported together. In the second survey period, “body aches” and “fatigue” were also commonly reported with other symptoms. Results from the phi-coefficient analysis for pairs of terms are shown in Supplementary Material 6. In the first survey period, “injection” and “pain” showed the highest correlation coefficients, followed by “abdominal pain” and “diarrhea,” and then “cough” and “sputum.” In the second survey period, the pair “injection” and “pain” again showed the strongest correlation.

## DISCUSSION

Using a text mining approach, we identified various common (≥1%) adverse reactions among individuals vaccinated against COVID-19 over 2 survey seasons, including reactions not commonly reported in the existing literature. Furthermore, the reporting percentages for most identified adverse reactions remained consistent, regardless of concurrent influenza vaccination.

In early 2021, COVID-19 vaccination campaigns began across multiple countries, including the United States [23]. The efficacy of these vaccines has been demonstrated through both randomized controlled trials and observational studies [31]. However, given the unprecedented speed of vaccine approval, our understanding of potential adverse events remains incomplete. Despite the passage of considerable time, COVID-19 vaccination programs and safety monitoring continue, aiming to address emerging variants and prevent outbreaks. Most studies have primarily focused on identifying a limited spectrum of severe adverse reactions associated with these vaccines. Text analytics provide an opportunity to uncover a broader range of both actual and potential concerns by analyzing keywords in large datasets [32]. Thus, we utilized text analysis to gain a more comprehensive understanding of vaccine safety. As a result, we identified pain in greater detail and captured a wide range of symptoms, including respiratory symptoms, sleep disturbances, lumbago, and indigestion.

Adverse reactions following vaccination can vary depending on the surveillance and reporting system, but local reactions, fatigue, headache, myalgia, and joint pain are commonly reported [11,12,33]. Sex-related and age-related differences in adverse event reporting have also been noted [11,34,35], potentially due to variations in immune response and behavioral factors [34]. However, direct comparisons with our study are limited by differences in the scope of reported reactions, vaccine types, and study populations. Some studies have reported temporary adverse events not previously documented in the literature. In line with earlier findings [15,36], symptoms related to colds were detected, as well as reactions such as sleep disturbance, indigestion, lumbago, and loss of appetite. Although earlier vaccine safety surveillance prompted investigations into symptoms like chest pain, dizziness, and generalized redness, our analysis revealed additional adverse reactions not previously emphasized [12].

Our text analysis also identified uncommon adverse reactions (≥0.1 to <1.0%) involving the eyes, ears, and oral cavity. Despite differences in vaccine types between seasons, a range of eye-related and ear-related symptoms was reported consistently across both years. Although no widespread vision-related adverse events have been reported for the COVID-19 vaccine, isolated cases of dry eyes and transient vision loss (amaurosis fugax) were described after the first and second doses of BNT162b2 mRNA (Pfizer-BioNTech, Comirnaty) in healthcare workers in Italy [34]. In China, monitoring of inactivated COVID-19 vaccines (WIV04 and HB02) found blurred vision in 2 patients after the second dose, and bloodshot or congested eyes in several cases; other uncommon reactions such as palpitations, oral infection, and irregular menstruation were also noted [35]. A study conducted in the United Kingdom among individuals vaccinated between December 8, 2020, and May 17, 2021, similarly reported adverse reactions involving the eyes and ears [37]. However, most reports focus only on local and systemic reactions, making it challenging to obtain information on various adverse reactions.

For practical reasons, the coadministration of the COVID-19 vaccine and the seasonal influenza vaccine was recommended [7,38]. Regarding the safety of simultaneous vaccination, previous studies found that individuals receiving both the seasonal influenza vaccine and a COVID-19 mRNA booster reported a slightly higher frequency of systemic reactions (8% with Pfizer-BioNTech booster and 11% with Moderna booster) compared to those who received only the COVID-19 vaccine, though these reactions were mild and transient [37,39]. The TACTIC study, a randomized clinical trial, showed that coadministration of influenza and COVID-19 vaccines resulted in lower quantitative and functional antibody responses compared to the COVID-19 vaccine alone [15]. However, no differences in adverse reactions were observed [15], which is consistent with previous findings [16,33].

In our study, during the 0-7 days after vaccination, sore throat and palpitations were reported more frequently among those who received only the COVID-19 vaccine compared to those who received both vaccines simultaneously; other adverse reactions were similar between groups. However, this pattern was not observed in the 2024-2025 survey. It is important to note that spontaneous reporting of adverse events enables cross-sectional assessment of actual experiences, but may result in underreporting or missing data [40,41]. Therefore, caution is warranted in drawing causal conclusions. Enhanced investigation systems may facilitate more comprehensive lists of adverse reactions, allowing differences to be verified in the future.

The advantage of text analysis lies in its ability to reveal prominent topics within text data and to clarify semantic relationships and meanings. We observed that reporting patterns for adverse reactions after vaccination varied over time, with cold-related symptoms increasing as time progressed (Figure 2). This may provide insights into temporal variations in symptom presentation. Our results are consistent with previous studies indicating that the pattern of symptom occurrence varies over time [37]. Reporting patterns also showed that pain-related and cold-related adverse reactions were frequently reported together (Figure 3), suggesting co-occurrence among vaccinated individuals. While 1 study described co-occurring COVID-19 symptoms using social media data [42], there is a lack of research on co-occurring adverse events after COVID-19 vaccination. Our analysis of text-based, self-reported data enabled the identification of a range of health problems by organ system and may provide further insight into potential adverse reactions.

When interpreting the results of this study, in addition to the limitations noted in previous research [3], several further considerations should be taken into account. Because responses frequently contained mixed descriptions, local and systemic adverse reactions were not analyzed separately. This may be due to unclear wording of survey questions or variations in participant understanding. Additionally, because symptoms were not verified using clinical diagnostic criteria, there may be concerns regarding the accuracy of reported adverse reactions. As data were collected through self-report, issues such as underreporting and missing data are possible. Since only text responses were analyzed, adverse reactions captured through closed-ended questions may be underestimated. However, most respondents answered both the closed-ended questions and provided text responses, likely resulting in some redundancy. Despite extensive efforts in preprocessing for text analysis, the accuracy of results may have been affected by inaccurate or irrelevant responses, as well as reports reflecting underlying medical history rather than new adverse events. Furthermore, because the survey primarily included older adults and immunocompromised individuals prioritized by the national immunization plan, findings should be interpreted in the context of this specific population. Caution is also warranted, as results are limited to respondents who consented to text message-based surveillance, were not linked to electronic health records, and may not capture the full spectrum of adverse events, reflecting only short-term reactions within the first 7 days after vaccination.

Nevertheless, we were able to explore in detail potential adverse reactions beyond those previously documented by employing a text mining approach. Analysis of data from both survey seasons revealed consistent reporting of respiratory symptoms, sleep disturbances, lumbago, and indigestion, as well as a variety of eye, ear, and oral cavity-related adverse reactions following COVID-19 vaccination. These findings highlight the need for ongoing and vigilant monitoring of such reactions after vaccination. We also investigated differences in adverse reactions reported in text responses according to the coadministration of influenza vaccine. This approach provided a broader understanding of concurrent adverse reactions, as visualized through continuous phi-coefficient and co-occurrence analyses.

Timely public health responses to safeguard population health are especially crucial during a pandemic. Active surveillance systems for vaccine safety that communicate directly with vaccine recipients allow for the rapid detection of safety signals. However, these systems also face the challenge of identifying potentially hidden adverse reactions within free-text data. As such, it is necessary to develop automated systems for classifying text-based data, and the findings of this study may serve as a foundation for such efforts.

This study explored adverse reactions based on self-reported symptoms using text mining of data collected through an active surveillance system for COVID-19 vaccines. Notably, commonly identified adverse reactions included respiratory symptoms, sleep disturbances, lumbago, and indigestion. Our findings contribute to a deeper understanding of vaccine adverse reactions and provide valuable information for improving future adverse event data management and surveillance systems.

## Figures and Tables

**Figure f4-epih-47-e2025034:**
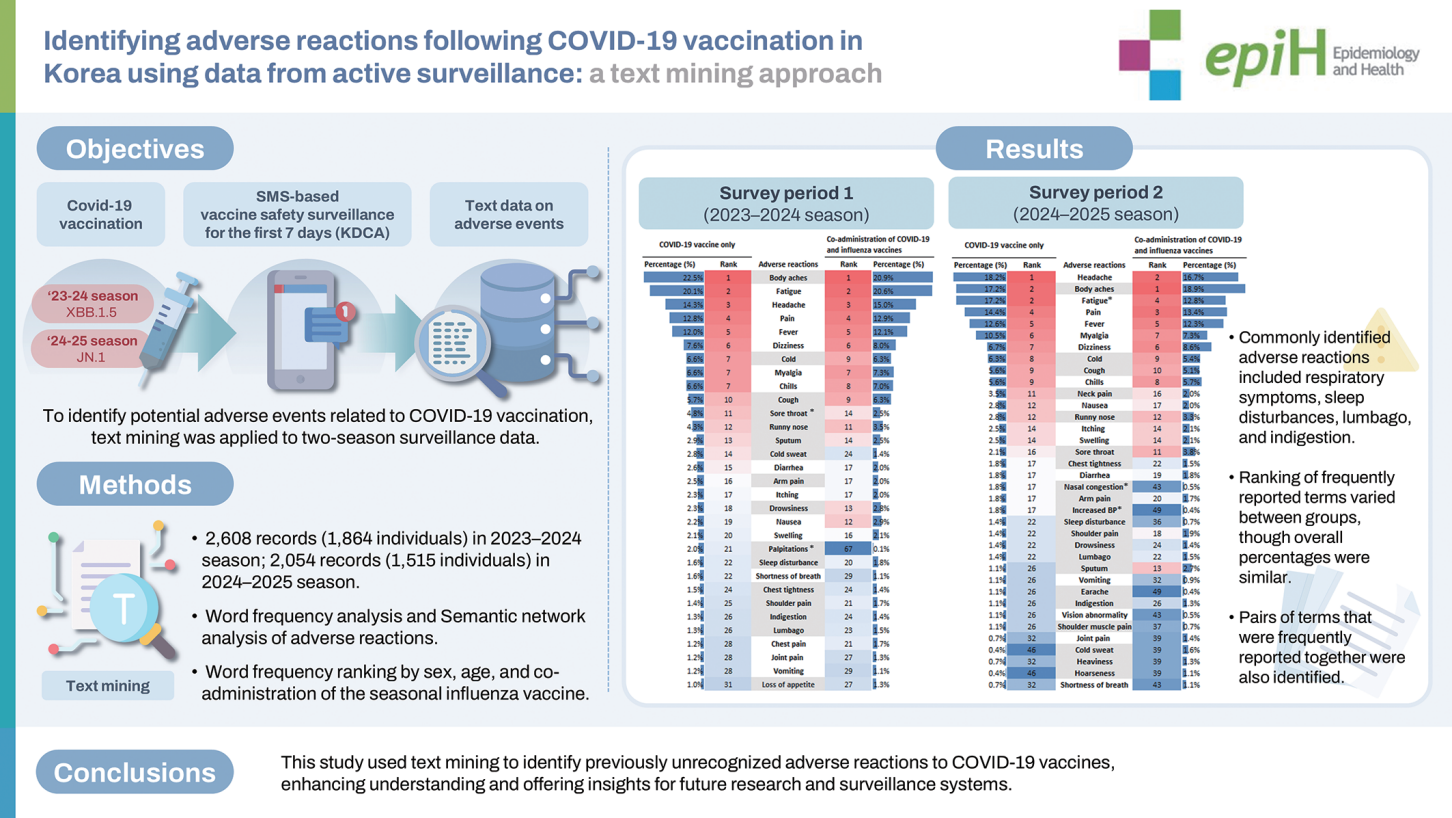


**Table 1.** Characteristics of study subjects who reported adverse reactions via text after COVID-19 vaccination

Characteristics	Total	COVID-19 vaccine only	Coadministration of COVID-19 and influenza vaccines	p-value
Survey period 1 (Oct 19, 2023 to Nov 6, 2023; n=1,864)				
Overall	1,864 (100)	1,152 (61.8)	712 (38.2)	
Sex				
Male	1,224 (65.7)	743 (64.5)	481 (67.6)	0.177
Female	640 (34.3)	409 (35.5)	231 (32.4)	
Age, mean±SD (yr)	68.11±12.03	67.17±12.53	69.63±11.01	<0.001
<65	360 (19.3)	260 (22.6)	100 (14.0)	<0.001
65-74	1,019 (54.7)	617 (53.5)	402 (56.5)	
≥75	485 (26.0)	275 (23.9)	210 (29.5)	
Vaccine manufacturer				
Moderna	1,022 (54.8)	580 (50.3)	442 (62.1)	<0.001
Pfizer-BioNTech	842 (45.2)	572 (49.6)	270 (37.9)	
Frequency of reports				
Mean± (SD)	1.40±0.83	1.41±0.84	1.39±0.81	0.598
Median (IQR)	1.00 (1.00-2.00)	1.0 (1.00-2.00)	1.00 (1.00-1.00)	0.564
Survey period 2 (Oct 11, 2024 to Nov 30, 2024; n=1,515)				
Overall	1,515 (100)	285 (18.8)	1,230 (81.2)	
Sex				
Male	954 (63.0)	168 (58.9)	786 (63.9)	0.119
Female	561 (37.0)	117 (41.0)	444 (36.1)	
Age (yr)	70.98±7.04	71.07±9.19	70.96±6.44	0.848
<65	61 (4.0)	33 (11.6)	28 (2.3)	<0.001
65-74	1,050 (69.3)	155 (54.4)	895 (72.8)	
≥75	404 (26.7)	97 (34.0)	307 (25.0)	
Vaccine manufacturer				
Moderna	674 (44.5)	114 (40.0)	560 (45.5)	<0.001
Pfizer-BioNTech	658 (43.4)	111 (38.9)	547 (44.5)	
Novavax	183 (12.1)	60 (21.0)	123 (10.0)	
Frequency of reports				
Mean±SD	1.36±0.75	1.41±0.80	1.34±0.74	0.201
Median (IQR)	1.00 (1.00-1.00)	1.00 (1.00-2.00)	1.00 (1.00-1.00)	0.098

Values are presented as number (%). COVID-19, coronavirus disease 2019; SD, standard deviation; IQR, interquartile range.

**Table 2.** Terms frequently reported as adverse reactions during the first 7 days following coronavirus disease 2019 vaccination

Category	Survey period 1 (Oct 19, 2023 to Nov 6, 2023)			Survey period 2 (Oct 11, 2024 to Nov 30, 2024)		
	Rank	Adverse reactions^1^	Frequency (%)^2^	Rank	Adverse reactions^1^	Frequency (%)^2^
Very common (≥10.0%)	1	Body aches	408 (21.9)	1	Body aches	281 (18.5)
	2	Fatigue	379 (20.3)	2	Headache	257 (17.0)
	3	Headache	272 (14.6)	3	Fatigue	207 (13.7)
	4	Pain	240 (12.9)	4	Pain	206 (13.6)
	5	Fever	224 (12.0)	5	Fever	187 (12.3)
Common (≥1.0 to <10.0%)	6	Dizziness	144 (7.7)	6	Dizziness	125 (8.3)
	7	Myalgia	128 (6.9)	7	Myalgia	120 (7.9)
	8	Chills	126 (6.8)	8	Chills	86 (5.7)
	9	Cold	121 (6.5)	9	Cold	84 (5.5)
	10	Cough	111 (6.0)	10	Cough	79 (5.2)
	11	Runny nose	75 (4.0)	11	Sore throat	53 (3.5)
	12	Sore throat	73 (3.9)	12	Runny nose	49 (3.2)
	13	Sputum	51 (2.7)	13	Sputum	36 (2.4)
	14	Nausea	46 (2.5)	14	Neck pain	35 (2.3)
	14	Drowsiness	46 (2.5)	15	Itching	33 (2.2)
	16	Diarrhea	44 (2.4)	15	Swelling	33 (2.2)
	17	Arm pain	43 (2.3)	17	Nausea	32 (2.1)
	18	Cold sweat	42 (2.3)	18	Diarrhea	27 (1.8)
	19	Itching	41 (2.2)	18	Shoulder pain	27 (1.8)
	20	Swelling	39 (2.1)	20	Arm pain	26 (1.7)
	21	Sleep disturbance	31 (1.7)	21	Chest tightness	23 (1.5)
	22	Shoulder pain	28 (1.5)	22	Lumbago	22 (1.5)
	23	Chest tightness	27 (1.4)	23	Cold sweat	21 (1.4)
	24	Chest pain	26 (1.4)	23	Drowsiness	21 (1.4)
	24	Lumbago	26 (1.4)	25	Joint pain	19 (1.3)
	24	Shortness of breath	26 (1.4)	25	Indigestion	19 (1.3)
	27	Indigestion	25 (1.3)	27	Heaviness	18 (1.2)
	28	Palpitations	24 (1.3)			
	29	Joint pain	23 (1.2)			
	30	Vomiting	22 (1.2)			
	31	Loss of appetite	20 (1.1)			

1 If an individual reported the same adverse reaction repeatedly over several days, it was treated as a single event; Gray background indicates newly identified adverse reactions via text analysis.

2 Percentage was calculated for 1,864 (survey period 1) and 1,515 (survey period 2) individuals.

## Supplementary materials

Supplementary Material 1.

Supplementary Material 2. Frequency and of reporting of text-based adverse reactions during the first 7 days following COVID-19 vaccination, according to whether the COVID-19 vaccine and seasonal influenza vaccine were administered at same time: (A) survey period 1 (October 19, 2023 to November 6, 2023) and (B) survey period 2 (October 11, 2024 to November 30, 2024)

Supplementary Material 3. Terms (below 1%) reported as adverse reactions during the first 7 days following COVID-19 vaccination

Supplementary Material 4. Differences of frequent terms for adverse reactions reported via text during the first 7 days following COVID-19 vaccination, according to sex: (A) survey period 1 (October 19, 2023 to November 6, 2023) and (B) survey period 2 (October 11, 2024 to November 30, 2024)

Supplementary Material 5. Differences of frequent terms for adverse reactions reported via text during the first 7 days following COVID-19 vaccination, according to age groups: (A) survey period 1 (October 19, 2023 to November 6, 2023) and (B) survey period 2 (October 11, 2024 to November 30, 2024)

Supplementary Material 6. Phi-coefficient network diagram for terms related to adverse reactions reported via text during the first 7 days following COVID-19 vaccination according to survey period: (A) survey period 1 (October 19, 2023 to November 6, 2023) and (B) survey period 2 (October 11, 2024 to November 30, 2024)
