# Supplementary material for: Identifying adverse reactions following COVID-19 vaccination in Korea using data from active surveillance: a text mining approach
Source: Epidemiol Health. 2025 Jun 30;47:e2025034. doi: 10.4178/epih.e2025034 (PMC12425858; doi:10.4178/epih.e2025034)
Supplement: Supplementary Material 1. [file epih-47-e2025034-Supplementary-1.docx]

**Supplementary Material 1**

***2*.*2 Data pre-processing for text-mining***

Text preprocessing included removing extra spaces, numbers, punctuation, correcting spelling errors and nouns, translating from English to Korean, tokenization, stopping word removal, and synonym processing. The ‘KoNLP’ package in R was utilized for tokenization and other preprocessing tasks. As stop words, directional terms (e.g., 'right', 'left', 'both'), time-related terms (e.g., 'morning', 'afternoon', 'evening'), and symptom severity terms (e.g., 'slightly', 'very', 'extremely', 'severely') were removed. Additionally, specific terms such as 'Tylenol' (a medication brand name) were excluded from the analysis to maintain focus on symptoms and adverse reactions. Due to the unique structure of the Korean language, where affixes form words and various verb derivations are common, expressions are often modified to nouns to reduce ambiguity. For instance, the phrase ‘I’m having a hard time with back pain’ is converted into ‘lumbago.’ For synonym processing, various symptom expressions were mapped to a common term. For instance, ‘difficulty speaking’ and ‘voice sounds hoarse’ were grouped under ‘Hoarseness’, while ‘pain when swallowing’ and ‘throat pain or a burning sensation in the throat’ were grouped under ‘Sore throat’. In addition, responses indicating symptom improvement, rather than adverse reactions, were excluded. Initial analysis also showed significant overlap between terms describing injection site reactions and systemic adverse reactions, so they were analyzed together in subsequent stages. The refined data from preprocessing facilitated word frequency analysis, calculation of daily reporting rates for prevalent terms, and semantic network analysis.

***2*.*3 Data analysis***

*2*.*3*.*2 Word frequencies*

In word-frequency analysis, to prevent exaggeration from individuals who responded multiple times (either across multiple days or within the same day), duplicate terms in an individual’s response were treated as omissions, and ‘injection’ was designated as a stop word. This approach allowed for the calculation of reporting percentages for 1,864 and 1,515 respondents, according to each survey period. Among the extracted terms, those related to adverse reactions reported by >1% of all respondents were ranked according to frequency. Furthermore, word frequency analysis was performed taking into consideration factors such as the coadministration of the influenza vaccine, sex, and age groups. Age groups were defined in detail considering the age distribution of respondents in each survey period (<65 years, 65–74 years, and ≥75 years for survey period 1; <75 years and ≥75 years for survey period 2). The daily adverse reaction reporting percentage was calculated to deduce when and for how long adverse reactions occurred after vaccination. Analysis was conducted for each day (from day 0–7) during the survey period. If a respondent reported the same term multiple times within a day, it was considered a duplicate term and treated as missing, and word frequency was calculated accordingly. Results, focusing on the top 10 most frequently reported terms daily, were organized and visualized for clear insight into the trends of adverse reactions.
