# Supplementary material for: Identifying adverse reactions following COVID-19 vaccination in Korea using data from active surveillance: a text mining approach
Source: Epidemiol Health. 2025 Jun 30;47:e2025034. doi: 10.4178/epih.e2025034 (PMC12425858; doi:10.4178/epih.e2025034)
Supplement: Supplementary Material 2. — Frequency and of reporting of text-based adverse reactions during the first 7 days following COVID-19 vaccination, according to whether the COVID-19 vaccine and seasonal influenza vaccine were administered at same time: (A) survey period 1 (October 19, 2023 to November 6, 2023) and (B) survey period 2 (October 11, 2024 to November 30, 2024) [file epih-47-e2025034-Supplementary-2.docx]

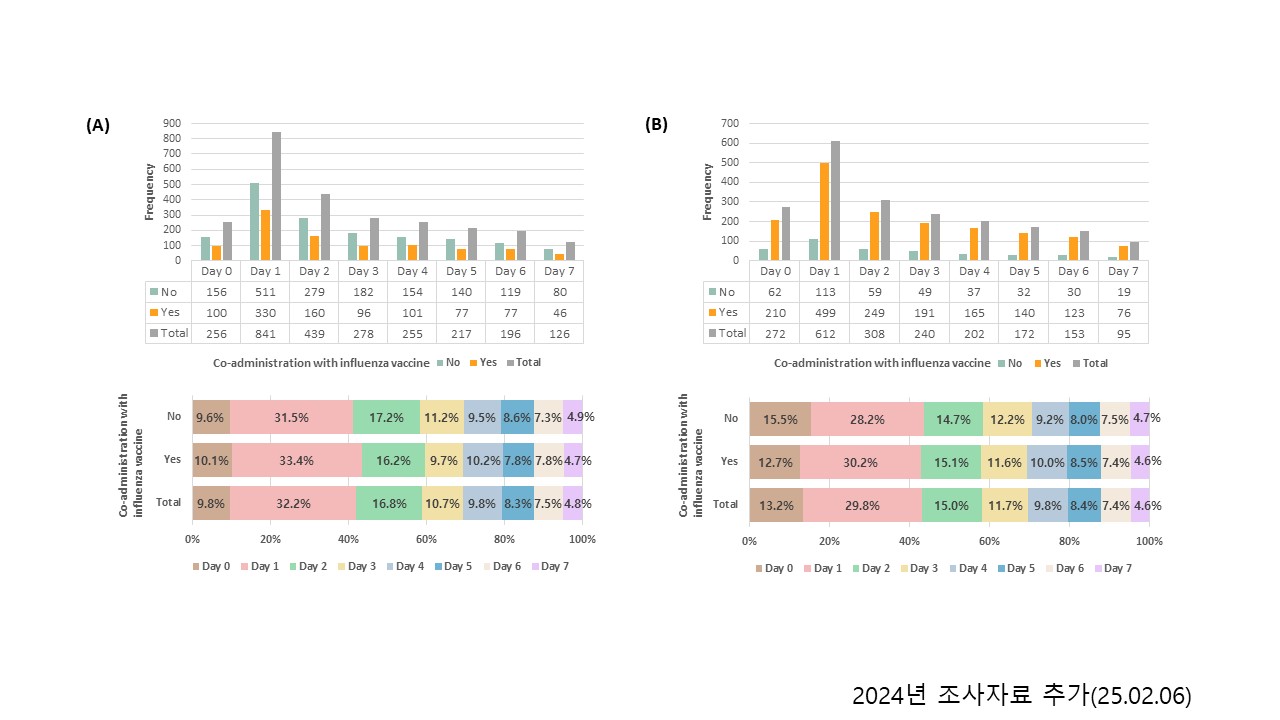


**Supplementary Material 2.** Frequency and of reporting of text-based adverse reactions during the first 7 days following COVID-19 vaccination, according to whether the COVID-19 vaccine and seasonal influenza vaccine were administered at same time: (A) survey period 1 (October 19, 2023 to November 6, 2023) and (B) survey period 2 (October 11, 2024 to November 30, 2024)

The upper plot shows daily reporting frequency after vaccination and the lower plot shows the daily reporting percentage after vaccination.
