# Supplementary material for: Identifying adverse reactions following COVID-19 vaccination in Korea using data from active surveillance: a text mining approach
Source: Epidemiol Health. 2025 Jun 30;47:e2025034. doi: 10.4178/epih.e2025034 (PMC12425858; doi:10.4178/epih.e2025034)
Supplement: Supplementary Material 3. — Terms (below 1%) reported as adverse reactions during the first 7 days following COVID-19 vaccination [file epih-47-e2025034-Supplementary-3.docx]

**Supplementary Material 3.** Terms (below 1%) reported as adverse reactions during the first 7 days following COVID-19 vaccination

|  | **Survey period 1**  (October 19, 2023 to November 6, 2023) | | **Survey period 2**  (October 11, 2024 to November 30, 2024) | |
| --- | --- | --- | --- | --- |
| Category | Adverse reactions | Frequency (%) | Adverse reactions | Frequency (%) |
| Uncommon  (≥ 0.1% to < 1.0%) | Abdominal pain | 16 (0.9%) | Shortness of breath | 15 (1%) |
|  | Vision abnormality | 15 (0.8%) | Vomiting | 14 (0.9%) |
|  | Hoarseness | 13 (0.7%) | Abdominal pain | 14 (0.9%) |
|  | Nasal congestion | 13 (0.7%) | Hoarseness | 14 (0.9%) |
|  | Leg pain | 12 (0.6%) | Loss of appetite | 14 (0.9%) |
|  | Discomfort in arm movement | 12 (0.6%) | Sleep disturbance | 13 (0.9%) |
|  | Shoulder muscle pain | 11 (0.6%) | Chest pain | 12 (0.8%) |
|  | Blushing | 11 (0.6%) | Palpitations | 11 (0.7%) |
|  | Sneezing | 10 (0.5%) | Shoulder muscle pain | 11 (0.7%) |
|  | Whole body | 9 (0.5%) | Nasal congestion | 11 (0.7%) |
|  | Rib pain | 8 (0.4%) | Leg | 10 (0.7%) |
|  | Numbness in legs | 8 (0.4%) | Increased BP | 10 (0.7%) |
|  | Brain fog | 8 (0.4%) | Vision abnormality | 9 (0.6%) |
|  | Knee pain | 8 (0.4%) | Earache | 8 (0.5%) |
|  | Inconvenience | 8 (0.4%) | Leg pain | 8 (0.5%) |
|  | Finger pain | 8 (0.4%) | Whole body | 8 (0.5%) |
|  | Tinnitus | 8 (0.4%) | Stomatitis | 7 (0.5%) |
|  | Leg muscle pain | 7 (0.4%) | Eye congestion | 7 (0.5%) |
|  | Leg weakness | 7 (0.4%) | Brain fog | 7 (0.5%) |
|  | Knee | 7 (0.4%) | Sneezing | 7 (0.5%) |
|  | Eye pain | 7 (0.4%) | Whole body pain | 7 (0.5%) |
|  | Increased blood pressure | 7 (0.4%) | Blushing | 7 (0.5%) |
|  | Earache | 6 (0.3%) | Bruise | 6 (0.4%) |
|  | Eye congestion | 6 (0.3%) | Tinnitus | 6 (0.4%) |
|  | Bone joint | 6 (0.3%) | Ear fullness | 6 (0.4%) |
|  | Facial edema' | 6 (0.3%) | Coryza | 6 (0.4%) |
|  | Difficulty moving | 5 (0.3%) | Discomfort in arm movement | 6 (0.4%) |
|  | Bruise | 5 (0.3%) | Leg weakness | 5 (0.3%) |
|  | Loss of taste | 5 (0.3%) | Eye discomfort | 5 (0.3%) |
|  | Eye discomfort | 5 (0.3%) | Shoulder | 5 (0.3%) |
|  | Nosebleed | 5 (0.3%) | Waist | 5 (0.3%) |
|  | Waist | 5 (0.3%) | Knee pain | 4 (0.3%) |
|  | Thighs | 5 (0.3%) | Finger pain | 4 (0.3%) |
|  | Armpit pain | 4 (0.2%) | Shoulder discomfort | 4 (0.3%) |
|  | Stomatitis | 4 (0.2%) | Face | 4 (0.3%) |
|  | Neck discomfort | 4 (0.2%) | Dry mouth | 4 (0.3%) |
|  | Heaviness | 4 (0.2%) | Lip blisters | 4 (0.3%) |
|  | Menstruation | 4 (0.2%) | Lower body | 4 (0.3%) |
|  | Itchy face | 4 (0.2%) | Numbness | 3 (0.2%) |
|  | Side | 4 (0.2%) | Difficulty moving | 3 (0.2%) |
|  | Gum pain | 4 (0.2%) | Numbness in legs | 3 (0.2%) |
|  | Memory impairments | 3 (0.2%) | Skin flare | 3 (0.2%) |
|  | Tear | 3 (0.2%) | Rash | 3 (0.2%) |
|  | Hives | 3 (0.2%) | Abdominal distension | 3 (0.2%) |
|  | Itchy scalp | 3 (0.2%) | Side | 3 (0.2%) |
|  | Motion Sickness | 3 (0.2%) | Nosebleed | 3 (0.2%) |
|  | Neck pain | 3 (0.2%) | Back muscle pain | 3 (0.2%) |
|  | Toe pain | 3 (0.2%) | Eyelid | 2 (0.1%) |
|  | Rash | 3 (0.2%) | Blepharospasm | 2 (0.1%) |
|  | Abdominal distension | 3 (0.2%) | Tear | 2 (0.1%) |
|  | Red spot | 3 (0.2%) | Eye fatigue | 2 (0.1%) |
|  | Irregular menstruation | 3 (0.2%) | Shingles | 2 (0.1%) |
|  | Hot eye | 3 (0.2%) | Neck discomfort | 2 (0.1%) |
|  | Shoulder | 3 (0.2%) | Knee | 2 (0.1%) |
|  | Shoulder joint pain | 3 (0.2%) | Ankle | 2 (0.1%) |
|  | Shoulder heavy | 3 (0.2%) | Wrist | 2 (0.1%) |
|  | Ear fullness | 3 (0.2%) | Shoulder heavy | 2 (0.1%) |
|  | Dry mouth | 3 (0.2%) | Hip | 2 (0.1%) |
|  | Back muscle pain | 3 (0.2%) | Urethral pain | 2 (0.1%) |
|  | Numbness | 2 (0.1%) | Lip | 2 (0.1%) |
|  | Armpit itching | 2 (0.1%) | Dead skin cells on lips | 2 (0.1%) |
|  | Earache | 2 (0.1%) | Lip Swelling | 2 (0.1%) |
|  | Leg | 2 (0.1%) | Periodontitis | 2 (0.1%) |
|  | Difficulty moving | 2 (0.1%) | Gum pain | 2 (0.1%) |
|  | Leg swelling | 2 (0.1%) | Limb muscles | 2 (0.1%) |
|  | Stuffy | 2 (0.1%) |  |  |
|  | Back muscle pain | 2 (0.1%) |  |  |
|  | Neck muscle pain | 2 (0.1%) |  |  |
|  | Ankle pain | 2 (0.1%) |  |  |
|  | Sole pain | 2 (0.1%) |  |  |
|  | Unrest | 2 (0.1%) |  |  |
|  | Upper body itching | 2 (0.1%) |  |  |
|  | Wrist pain | 2 (0.1%) |  |  |
|  | Shoulder discomfort | 2 (0.1%) |  |  |
|  | Hip | 2 (0.1%) |  |  |
|  | Dead skin cells on lips | 2 (0.1%) |  |  |
|  | Lip blisters | 2 (0.1%) |  |  |
|  | Gum swelling | 2 (0.1%) |  |  |
|  | Tooth pain | 2 (0.1%) |  |  |
|  | Itchy arms | 2 (0.1%) |  |  |
|  | Elbow | 2 (0.1%) |  |  |
|  | Tonsil edema | 2 (0.1%) |  |  |
|  | Tonsil | 2 (0.1%) |  |  |

If an individual reported the same adverse reaction repeatedly over several days, it was treated as a single event. Percentage (%) was calculated for 1,864 (survey period 1) and 1,515 (survey period 2) individuals.
