# Supplementary material for: Identifying adverse reactions following COVID-19 vaccination in Korea using data from active surveillance: a text mining approach
Source: Epidemiol Health. 2025 Jun 30;47:e2025034. doi: 10.4178/epih.e2025034 (PMC12425858; doi:10.4178/epih.e2025034)
Supplement: Supplementary Material 4. — Differences of frequent terms for adverse reactions reported via text during the first 7 days following COVID-19 vaccination, according to sex: (A) survey period 1 (October 19, 2023 to November 6, 2023) and (B) survey period 2 (October 11, 2024 to November 30, 2024) [file epih-47-e2025034-Supplementary-4.docx]

| **(A)** | 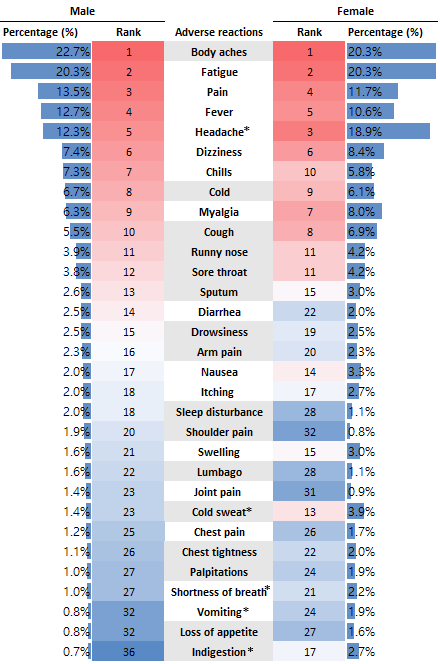 | **(B)** | 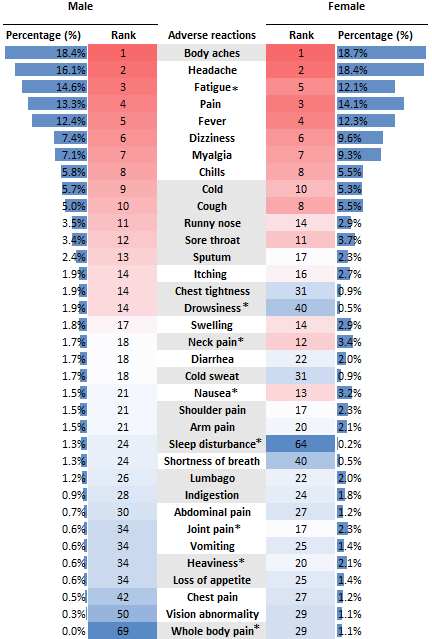 |
| --- | --- | --- | --- |

**Supplemental Material 4.** Differences of frequent terms for adverse reactions reported via text during the first 7 days following COVID-19 vaccination, according to sex: (A) survey period 1 (October 19, 2023 to November 6, 2023) and (B) survey period 2 (October 11, 2024 to November 30, 2024)

Asterisks indicate statistical differences (*p*<0.05) between groups.

Gray background indicates newly identified adverse reactions via text analysis.
