# Supplementary material for: Identifying adverse reactions following COVID-19 vaccination in Korea using data from active surveillance: a text mining approach
Source: Epidemiol Health. 2025 Jun 30;47:e2025034. doi: 10.4178/epih.e2025034 (PMC12425858; doi:10.4178/epih.e2025034)
Supplement: Supplementary Material 5. — Differences of frequent terms for adverse reactions reported via text during the first 7 days following COVID-19 vaccination, according to age groups: (A) survey period 1 (October 19, 2023 to November 6, 2023) and (B) survey period 2 (October 11, 2024 to November 30, 2024) [file epih-47-e2025034-Supplementary-5.docx]

| **(A)** | **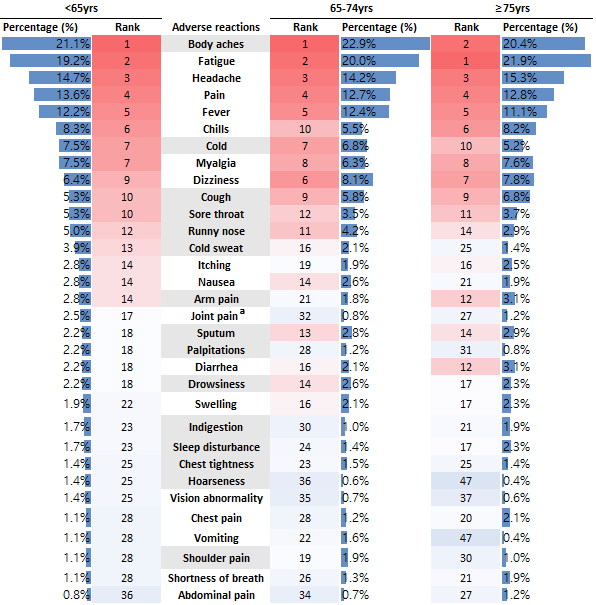** | **(B)** | **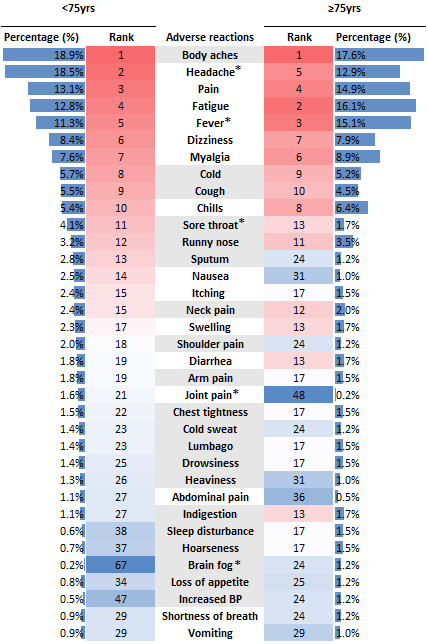** |
| --- | --- | --- | --- |

**Supplemental Material 5.** Differences of frequent terms for adverse reactions reported via text during the first 7 days following COVID-19 vaccination, according to age groups: (A) survey period 1 (October 19, 2023 to November 6, 2023) and (B) survey period 2 (October 11, 2024 to November 30, 2024)

The superscript letter 'a' indicates a statistical difference (Bonferroni adjusted *p*<0.05) between the group under 65 years of age and the group 65-74 years of age and asterisks in the right plot indicate statistical differences (*p*<0.05) between two groups.

Gray background indicates newly identified adverse reactions via text analysis.
